# Utility of Follow-Up Computed Tomography (CT) and Fluorodeoxyglucose-Positron Emission Tomography/CT (FDG-PET/CT) in Diagnosing Gastrointestinal Stromal Tumor (GIST) Presenting as Spontaneous Hemoperitoneum: A Case Report

**DOI:** 10.7759/cureus.69799

**Published:** 2024-09-20

**Authors:** Susumu Doita, Megumi Watanabe, Toshihiro Ogawa, Kohji Tanakaya, Hideki Aoki

**Affiliations:** 1 Department of Gastroenterological Surgery, Okayama University Graduate School of Medicine, Dentistry, and Pharmaceutical Sciences, Okayama, JPN; 2 Department of Surgery, National Hospital Organization Iwakuni Clinical Center, Iwakuni, JPN

**Keywords:** 18f-fluorodeoxyglucose positron emission tomography (18f-fdg pet), gastrointestinal stromal tumor (gist), massive hematoma, rare cause of acute abdominal pain, spontaneous hemoperitoneum

## Abstract

Spontaneous hemoperitoneum is a rare and potentially life-threatening condition with a wide differential diagnosis. Gastrointestinal stromal tumors (GIST) can present with spontaneous hemoperitoneum, although diagnosing GIST as the cause of hemoperitoneum is challenging due to its rarity.

A 76-year-old Japanese man presented with sudden epigastric pain and was found to have a 10 cm space-occupying lesion and ascites on ultrasonography. Despite stable vital signs, computed tomography (CT) findings showed a 10×15 cm mass with heterogeneously enhanced solid and cystic lesions, and the patient opted for conservative treatment. Two months later, a contrast-enhanced CT scan revealed a high-density area within the hematoma, prompting further investigation with fluorodeoxyglucose-positron emission tomography/CT (FDG-PET/CT), which showed FDG accumulation suggestive of malignancy. Exploratory laparotomy revealed a large encapsulated mass from the greater omentum, and histopathology confirmed a diagnosis of high-risk extraluminal gastric GIST. The patient was successfully treated with surgical resection.

This case highlights two important clinical issues. First, follow-up CT and FDG-PET/CT are useful in detecting GIST when an unexplained intraperitoneal hematoma is identified. Second, surgical intervention is recommended in such cases to determine the cause.

Contrast-enhanced follow-up CT and FDG-PET/CT are valuable in clarifying the presence of GIST, and surgical intervention is recommended to identify the causes of intraperitoneal hematoma. Further studies are needed to standardize the approach to spontaneous hematoma from GIST.

## Introduction

Spontaneous hemoperitoneum is a rare and potentially life-threatening condition [1]. Gastrointestinal stromal tumor (GIST) can present as spontaneous hemoperitoneum [2]. Owing to their rarity, the diagnosis of GIST as the cause of hemoperitoneum may be challenging. Very few imaging follow-ups have been performed. Herein, we present a case in which follow-up computed tomography (CT) and fluorodeoxyglucose-positron emission tomography/CT (FDG-PET/CT) were useful for confirming the presence of GIST.

## Case presentation

A 76-year-old Japanese man with sudden epigastric pain was admitted to a local hospital. The patient was referred for a 10 cm space-occupying lesion and ascites on ultrasonography (US). He had no history of trauma. However, the patient had a history of atrial fibrillation and was prescribed rivaroxaban.

**Figure 1:**
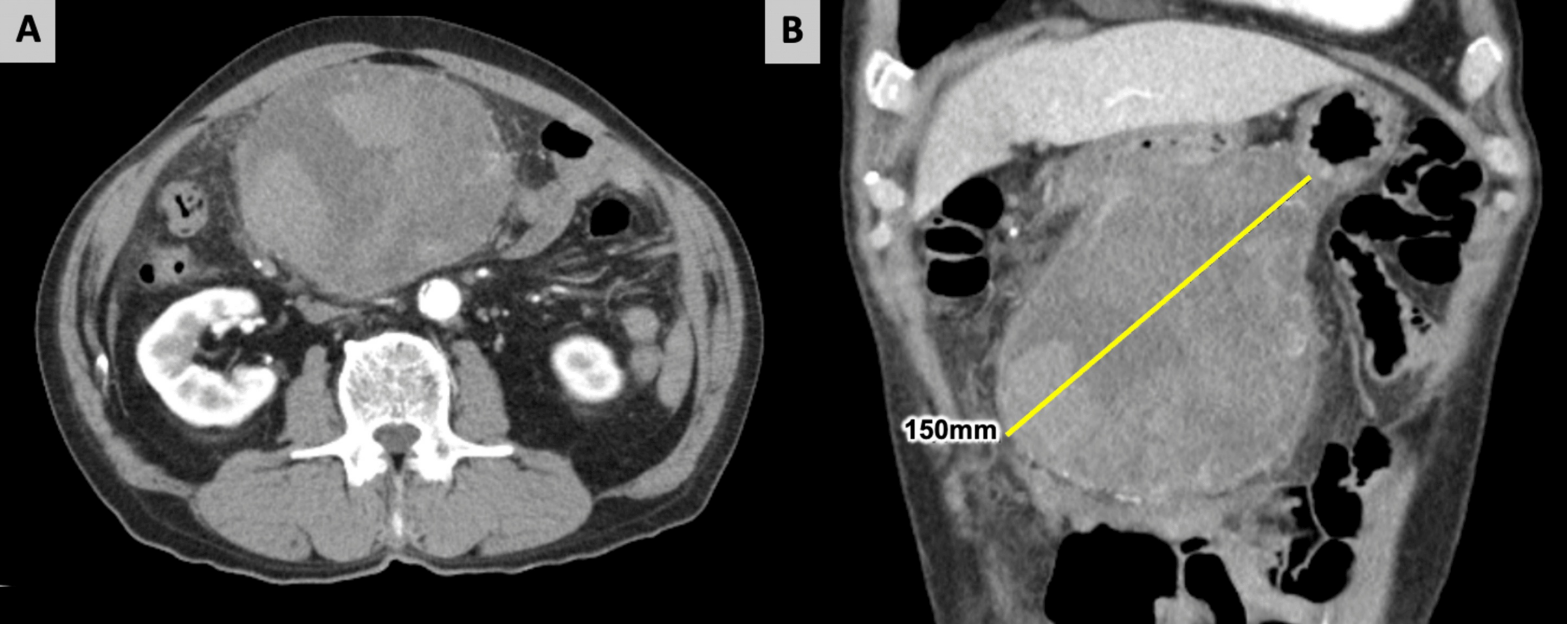
Contrast-enhanced abdominal CT on admission. (A) The axial view showed a huge mass consisting of heterogeneously enhanced solid and giant cystic lesions. (B) The coronal view showed a 100×150 mm mass. CT: computed tomography

Upon arrival at our hospital, the patient was conscious, and vital signs showed a heart rate of 98 bpm and atrial fibrillation, with a blood pressure of 123/67 mmHg. The patient experienced intermittent diffuse abdominal pain, nausea, and abdominal fullness. A head-sized mass with a smooth surface and good mobility was palpable in the mid-abdomen. Laboratory studies showed a low hemoglobin level of 10.7 g/dL, a white blood cell count of 5350/μL, a platelet count of 24.7×104/μL, an international normalized ratio of prothrombin time of 1.76, an activated partial thromboplastin time of 44.5 seconds, and a C-reactive protein level of 1.15 mg/dL. CT revealed a 100×150 mm mass consisting of heterogeneously enhanced solid and giant cystic lesions and ascites (Figure 1). However, we were unable to identify the source of the bleeding. The patient's vital signs were stable, and he opted for a conservative treatment. Although the hematoma size had not changed on US since admission, the patient's abdominal pain improved, and he was subsequently discharged after nine days.

**Figure 2:**
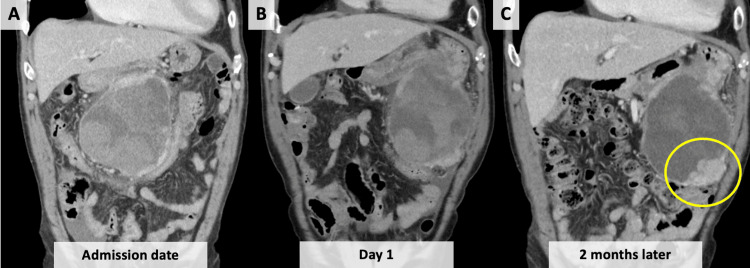
The course of contrast-enhanced abdominal CT scan findings revealing the size of the hematoma did not change. (A) Admission date. (B) Day 1. (C) Two months later. A part of the caudal side of the hematoma had a high-density area that had not been seen before (yellow circle). CT: computed tomography

**Figure 3:**
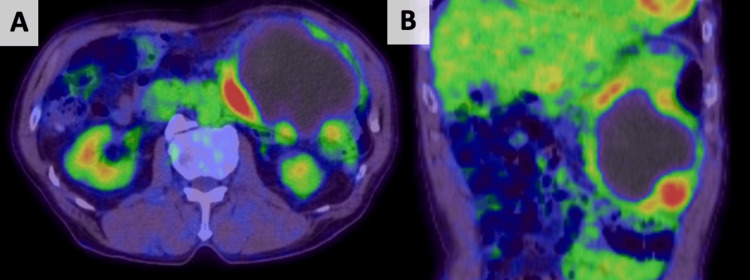
FDG-PET/CT scan showing the accumulation of FDG. (A) Axial view. (B) Coronal view. FDG-PET/CT: fluorodeoxyglucose-positron emission tomography/computed tomography

**Figure 4:**
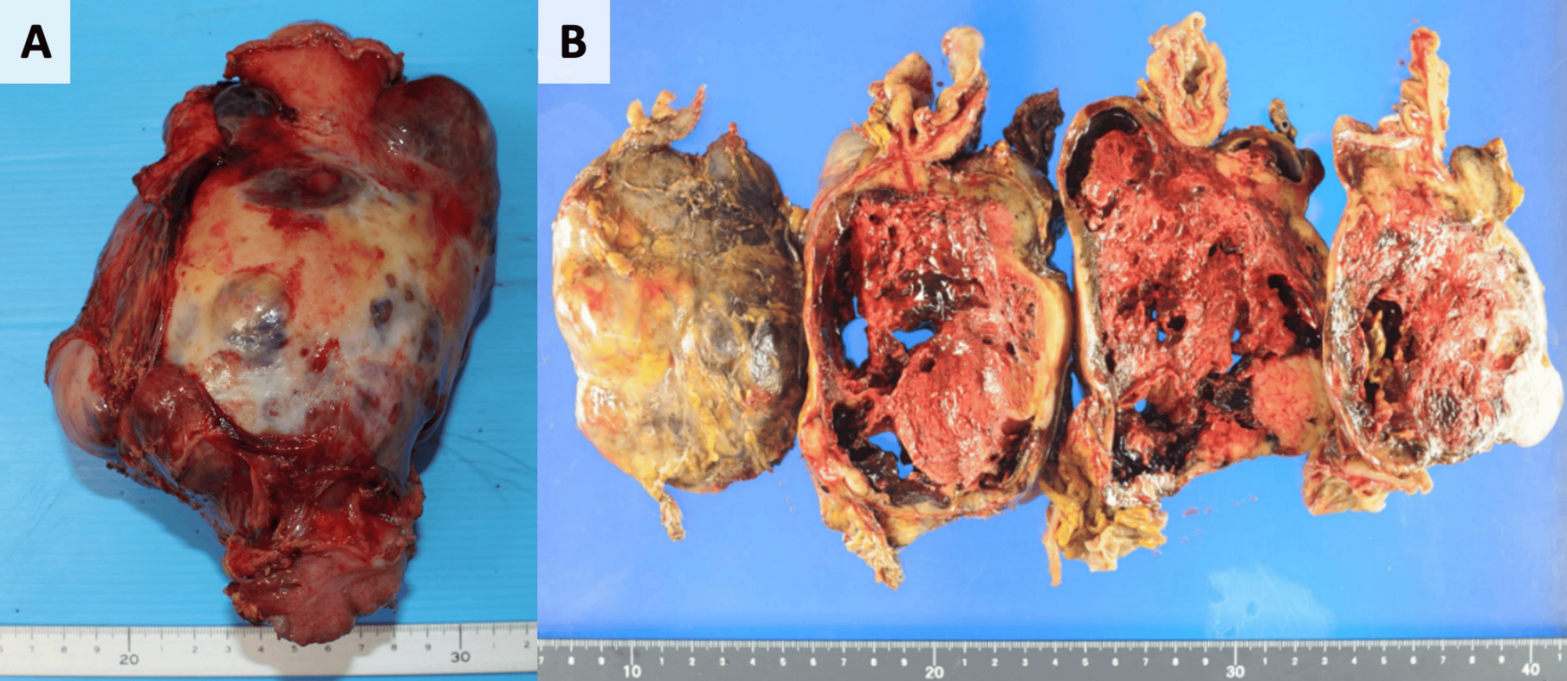
Gross examination findings. (A) Gross examination shows a globular soft tissue mass measuring 12.8×11×10 cm. (B) The cut surface of the specimen was a hematoma.

**Figure 5:**
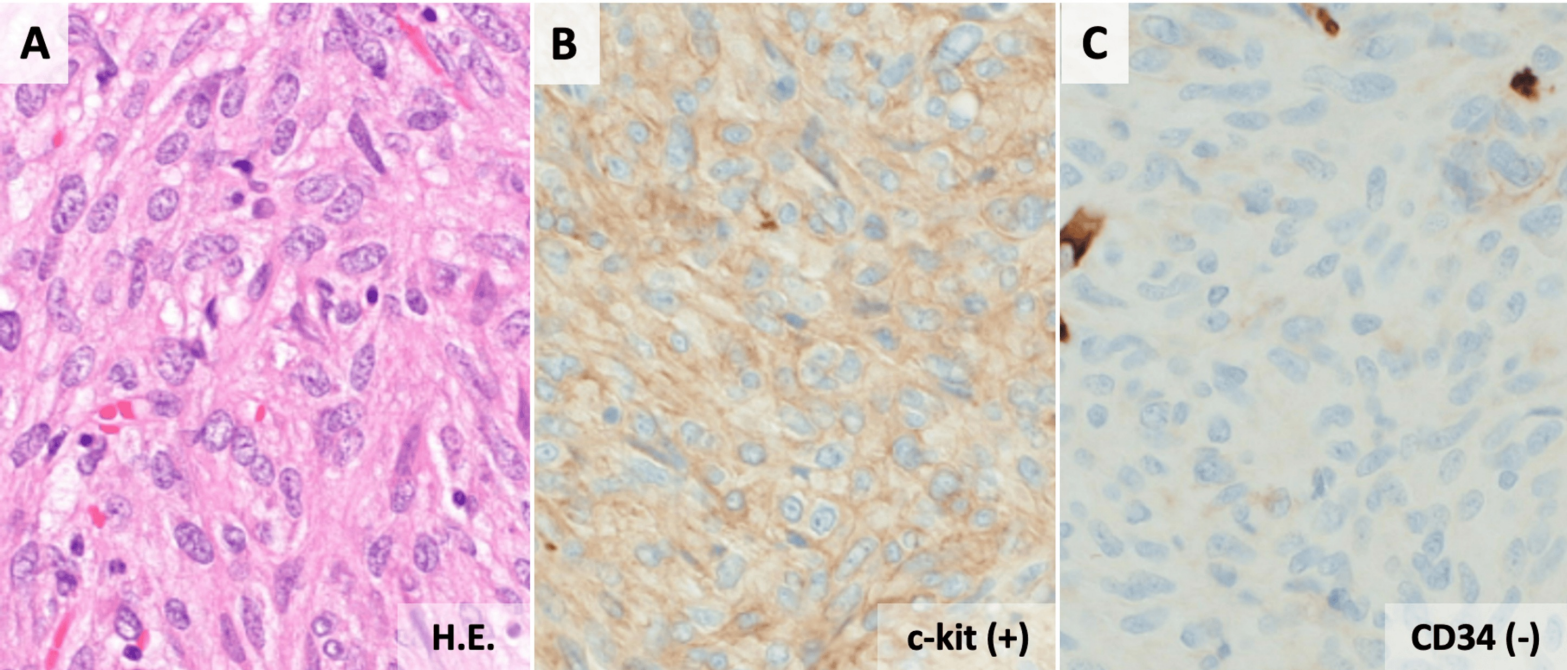
Pathological findings. (A) Microscopic examination (hematoxylin-eosin staining, original magnification: 200×) showed proliferation of spindle cells. Immunohistochemical staining revealed that tumor cells were positive for c-kit (B) and negative for CD34 (C).

Two months later, a contrast-enhanced CT scan showed that the size of the hematoma had not changed; however, a part of the caudal side of the hematoma had a high-density area that had not been observed before (Figure 2). Therefore, we suspected that the hematoma was caused by malignancy. Subsequently, we conducted FDG-PET/CT, which revealed FDG accumulation in that area (Figure 3). A spontaneous hematoma due to malignancy was strongly suspected, and the patient underwent exploratory laparotomy. Laparotomy revealed a large encapsulated mass (approximately 15 cm) arising from the greater omentum that adhered to the transverse colon and pylorus. Thus, an upper midline incision was made, and a combined resection of the adjacent organs was performed. Gross examination showed a globular soft tissue mass measuring 12.8×11×10 cm (Figure 4). The cut surface of the specimen was a hematoma. Histopathological examination revealed that the tumors consisted of proliferating spindle cells with massive necrosis (Figure 5A) and 16 mitoses per 50 consecutive high-power fields. Immunohistochemical examination revealed the expression of KIT (CD117) (Figure 5B) and was negative for CD34 (Figure 5C). Therefore, we made a diagnosis of high-risk extraluminal gastric GIST. The patient was discharged nine days after the operation and treated with imatinib mesylate. Two years after surgery, he presented to our hospital complaining of sudden epigastric pain. An abdominal CT scan revealed peritoneal metastasis and hemoperitoneum. The patient is currently undergoing palliative treatment.

## Discussion

Spontaneous hemoperitoneum is a rare and potentially life-threatening condition with a wide range of differential diagnoses (Table 1) [1]. GIST can present as spontaneous hemoperitoneum [2]. This case suggests that intraperitoneal hematomas from GIST have the following two characteristics: When an unexplained intraperitoneal hematoma is identified, follow-up CT and FDG-PET/CT are useful for identifying the presence of GIST, and surgical intervention is recommended to identify the causes.

**Table 1:** Differential diagnosis of spontaneous intraperitoneal hemorrhage. GIST: gastrointestinal stromal tumor

Organ system	Differential diagnosis
Gynecological	Ectopic pregnancy
	Ruptured ovarian cyst
	Uterine leiomyoma/leiomyosarcoma
	Ovarian granulosa cell tumor
	Ruptured corpus luteum cyst
	Hyperemesis gravidarum
	Endometriosis
	Spontaneous uterine rupture
Splenic	Chronic myelomonocytic leukemia
	Infectious mononucleosis
	Spontaneous splenic rupture
	Iatrogenic
	Spontaneous rupture of the splenic vein
	Torsion and rupture of wandering spleen
	Hamartoma
	Primary splenic angiosarcoma
Hepatic	Peliosis hepaticus
	Hepatic adenoma/adenomatosis
	Hepatocellular carcinoma
	Hemangioma
	Primary hepatic angiosarcoma
	Metastatic cancer
	Amyloid
	Focal nodular hyperplasia
Biliary	Ruptured cholangiocarcinoma
	Transhepatic rupture of the gallbladder
Vascular	Ruptured cystic artery pseudoaneurysm
	Ruptured splenic artery aneurysm
	Segmental mediolytic arteriopathy
Gastric	Mixed cavernous-capillary hemangioma
	GIST
Colon	Meckel's diverticulitis
	GIST
Pancreatic	Ruptured pseudocyst
Miscellaneous	Ruptured benign solitary fibrous tumor

First, we must be aware that GIST can be a cause of sudden intra-abdominal hemorrhage or hematoma. Additionally, follow-up CT and FDG-PET/CT are useful for determining the presence of malignant tumors. Therefore, we searched for previous reports on intraperitoneal hemorrhage caused by GIST in the PubMed database using the keywords "intraperitoneal hemorrhage, bleeding, or hematoma" and "GIST" and reviewed 24 cases. Most cases required emergency surgery, and very few cases required follow-up imaging. Typically, an intraperitoneal hematoma gradually becomes smaller as it is absorbed by the body, and the CT appearance of a hematoma can change from initial enhancement to non-enhancement as it resolves. However, we found that intraperitoneal hematomas from GIST have two characteristics. First, an intraperitoneal hematoma from a GIST does not become smaller because it is formed by a tumor. Second, the tumor becomes apparent over time. At the initial presentation, the relatively high density of the hematoma on CT was similar to that of the tumor, making the tumor not apparent. Gradually, as the hematoma density decreased, the tumor became apparent. In the present case, these findings were observed, and a tumor was suspected. FDG-PET/CT revealed FDG accumulation in that area. Thus, follow-up CT and FDG-PET/CT are useful for identifying the presence of GIST.

Second, surgical resection should be performed to identify the cause of spontaneous intraperitoneal hematoma when the cause is unclear. In previous reports on GIST, most patients with intraperitoneal bleeding from GIST had abdominal pain and hematoma on CT findings. These symptoms are similar to those of idiopathic omental bleeding [3]. Some previous reports on idiopathic omental bleeding have recommended conservative treatment, and in recent years, minimally invasive treatments, such as transcatheter arterial embolization (TAE) for idiopathic omental bleeding, have been reported and are preferred [4-6]. In cases of GIST, complete surgical resection is the only potentially curative treatment. Distinguishing between these conditions can be challenging because of their overlapping clinical features. Thus, surgical resection should be performed to identify the cause of spontaneous intraperitoneal hematoma.

Table 2 lists the previous reports of intraperitoneal hemorrhage caused by GIST.

**Table 2:** Previous reports of intraperitoneal hemorrhage caused by GIST. GIST: gastrointestinal stromal tumor

No.	Study	Age (years)	Sex	Symptom	Tumor size	Operation	Postoperative diagnosis
1	Hirasaki et al. (2008) [7]	87	M	Loss of consciousness	13 cm	Ileal resection	Ileal GIST
2	Bae and Kim (2009) [8]	33	M	Melena/hematochezia	NA	Distal gastrectomy	Gastric GIST
3	Fiscon et al. (2009) [9]	68	M	Abdominal pain	11 cm	NA	Gastric GIST
4	Enomoto et al. (2010) [10]	75	M	Abdominal pain	13 cm	Resection of the jejunum and the omentum	Jejunal GIST
5	Iusco et al. (2010) [11]	76	M	Abdominal pain/nausea	20 cm	Ileal resection	Ileal GIST
6	Sprenger et al. (2010) [12]	38	M	NA	NA	NA	Gastric GIST
7	Varras et al. (2010) [13]	28	F	Abdominal pain	13 cm	Small bowel resection	Small bowel GIST
8	Costi et al. (2011) [14]	81	M	Abdominal pain/anemia	4 cm	Sleeve gastrectomy	Gastric GIST
9	de Juan Ferré et al. (2012) [15]	62	F	Abdominal pain	NA	NA	NA
10	Murayama et al. (2012) [16]	43	M	Abdominal pain	19 cm	Omentectomy	Omental GIST
11	Yakan et al. (2012) [17]	51	M	Abdominal pain	6 cm	Partial gastrectomy	Gastric GIST
12	Seow-En et al. (2014) [18]	60	F	Abdominal pain	22 cm	NA	Omental GIST
13	Attaallah et al. (2015) [19]	46	M	Abdominal pain	8 cm	Small bowel resection	Jejunal GIST
14	Vinagreiro et al. (2015) [20]	65	M	No symptom	17 cm	Partial gastrectomy	Gastric GIST
15	Hosamani et al. (2016) [21]	40	M	Abdominal pain	4 cm	Meckel's diverticulum was resected	GIST
16	Islam et al. (2017) [22]	67	M	NA	NA	NA	Omental GIST
17	Sato et al. (2017) [23]	74	M	Abdominal pain/nausea	14 cm	Small bowel resection	Jejunal GIST
18	Fukuda et al. (2017) [24]	72	M	Abdominal pain	2 cm	Small bowel resection	Jejunal GIST
19	Shrestha and Shrestha (2018) [25]	28	F	Abdominal pain	7 cm	Ileal resection	Ileal GIST
20	Shively et al. (2020) [26]	63	F	Epigastric pain	7 cm	Partial gastrectomy	Gastric GIST
21	Arata et al. (2020) [27]	46	M	Abdominal pain	7 cm	Small bowel resection	Jejunal GIST
22	Almeida et al. (2022) [28]	79	M	Abdominal pain	10 cm	Partial gastrectomy	Gastric GIST
23	Abdelgawad et al. (2022) [29]	81	M	Abdominal pain/nausea	5 cm	Small bowel resection	Jejunal GIST
24	Meader et al. (2023) [30]	37	F	Abdominal pain	3 cm	Subtotal gastric resection	Gastric GIST
25	Present case (2024)	75	M	Abdominal pain	15 cm	Distal gastrectomy/partial colectomy	Gastric GIST

## Conclusions

This case report indicates that contrast-enhanced follow-up CT and FDG-PET/CT are useful for detecting the presence of GIST when faced with an unexplained intraperitoneal hematoma. These imaging modalities can help to differentiate between hematomas caused by benign conditions and those caused by malignant tumors, such as GIST. Furthermore, when the cause of a spontaneous intraperitoneal hematoma is unclear, surgical intervention is recommended to identify the underlying pathology. Future studies could contribute to standardizing the approach for spontaneous hematoma formation in GIST.
